# Preoperative Planning Using Three-Dimensional Multimodality Imaging for Soft Tissue Sarcoma of the Axilla: A Pilot Study

**DOI:** 10.3390/cancers14133185

**Published:** 2022-06-29

**Authors:** Xiang Fang, Yan Xiong, Fang Yuan, Senlin Lei, Dechao Yuan, Yi Luo, Yong Zhou, Li Min, Wenli Zhang, Chongqi Tu, Hong Duan

**Affiliations:** 1 Department of Orthopedics, Orthopedic Research Institute, West China Hospital, Sichuan University, Chengdu 610041, China; xiangfang@stu.scu.edu.cn (X.F.); luyibingli@163.com (Y.X.); lsl867172079@126.com (S.L.); lzyxyncszxyyydc@163.com (D.Y.); orthop_luoyi@163.com (Y.L.); changfshn@163.com (Y.Z.); jacky-min@163.com (L.M.); tcqbonetumor@163.com (C.T.); duanhong1970@126.com (H.D.); 2 Department of Radiology, West China Hospital, Sichuan University, Chengdu 610041, China; yuanfang@wchscu.cn

**Keywords:** axilla, soft tissue sarcoma, magnetic resonance neurography, surgical margin

## Abstract

**Simple Summary:**

Soft tissue sarcoma (STS) of the axilla, with its proximity to vital neurovascular bundles and occasional involvement, is a challenge for surgeons. Conventionally, surgeons need to build the whole tumour model with its adjacent anatomical structures by absorbing necessary information from each separate preoperative 2D and 3D image, which is very experience-demanding and potentially inaccurate. Therefore, a computer-generated 3D tumour model revealing tumour and adjacent key anatomical structures from multimodal images was developed, and we attempted to explore whether this digital model could facilitate surgical planning and outcomes for axillary STS. This study suggested significantly better performance in reducing surgical blood loss, operative time, and length of hospital stay. Considering that the surgeries were performed by two specialists with 15 years of experience, the real-world benefit might be even greater, especially for less-experienced STS surgeons. Therefore, this technology might change how preoperative planning is performed for complex STS in the future.

**Abstract:**

Axillary soft tissue sarcoma (STS) is challenging due to its proximity to vital neurovascular bundles. We conducted a prospective observational pilot study to explore whether 3D multimodality imaging (3DMMI) can improve preoperative planning for and surgical outcomes of patients with axillary STS. Twenty-one patients with STS (diameter > 5 cm) of the axilla were allocated, at their discretion, to either a control group undergoing traditional preoperative planning with separate computed tomography angiography, magnetic resonance imaging, and magnetic resonance neurography, or an intervention group where 3DMMI, digitally created based on these images, revealed the tumour and adjacent skeletomuscular and neurovascular structures in three dimensions. Primary outcome measures were surgical margins and surgical complications. Secondary outcomes included operative time, blood loss, serum C-reactive protein and interleukin-6, length of hospital stay, and limb function. The 3DMMI group had a lower, although not significantly different, inadvertent positive margin rate (1/12 vs. 3/9, *p* = 0.272), a significantly shorter operative time (*p* = 0.048), reduced blood loss (*p* = 0.038), and reduced length of hospital stay (*p* = 0.046). This endorses larger trials to improve complex surgical procedures and study how preoperative planning could be performed in the future.

## 1. Introduction

Soft tissue sarcomas (STS) encompass over 100 histologic subtypes, accounting for 1% of all adult malignancies [1]. In 2018, approximately 13,040 individuals were diagnosed with STS in the USA, with an estimated 5150 deaths [2]. The most common subtypes are leiomyosarcoma, liposarcoma, and undifferentiated pleomorphic sarcoma, whereas the most common primary sites are extremities (43%), trunk (13%), and retroperitoneum (7%) [3]. Despite a multidisciplinary approach, the cornerstone treatment is surgical resection, in which en bloc resection with wide surgical margins is standard procedure [4,5].

Axillary STS has unique management challenges due to its proximity to important neurovascular structures. This makes wide surgical margins almost impossible and increases the risk of functional morbidity. Appropriate preoperative planning by experienced surgeons may reduce the risk of margin contamination and neurovascular injury. However, only a few studies have reported surgical improvement from the perspective of preoperative planning through imaging modalities [6,7,8,9]. Magnetic resonance imaging (MRI) and magnetic resonance neurography (MRN) are used for local staging and preoperative planning, especially regarding tumour location and its adjacent neural structures, usually in two dimensions (2D), whereas computed tomography (CT) plays a role in revealing vascular structures and calcified lesions in three dimensions (3D). In conventional surgical planning, the surgeon relies on these mixed 2D and 3D images to construct the tumour and its adjacent anatomical structures in the brain, which might be inaccurate, requires a high cognitive load, and demands very high experience.

3D multimodality imaging (3DMMI) is generated by radiologists using image registration and segmentation algorithms based on each imaging modality, where different images synergistically contribute to a more reliable computer-generated objective tumour building. Currently, 3DMMI is applied in neurosurgery, radiation therapy, and bone oncology surgery, where is demonstrates promising efficacy in clinical practice [10,11,12,13]. However, no relevant STS studies have been performed. This study aimed to report a pilot real-world experience with 3DMMI for the surgical treatment of axillary STS.

## 2. Materials and Methods

### 2.1. Study Design and Eligibility

**Figure 1:**
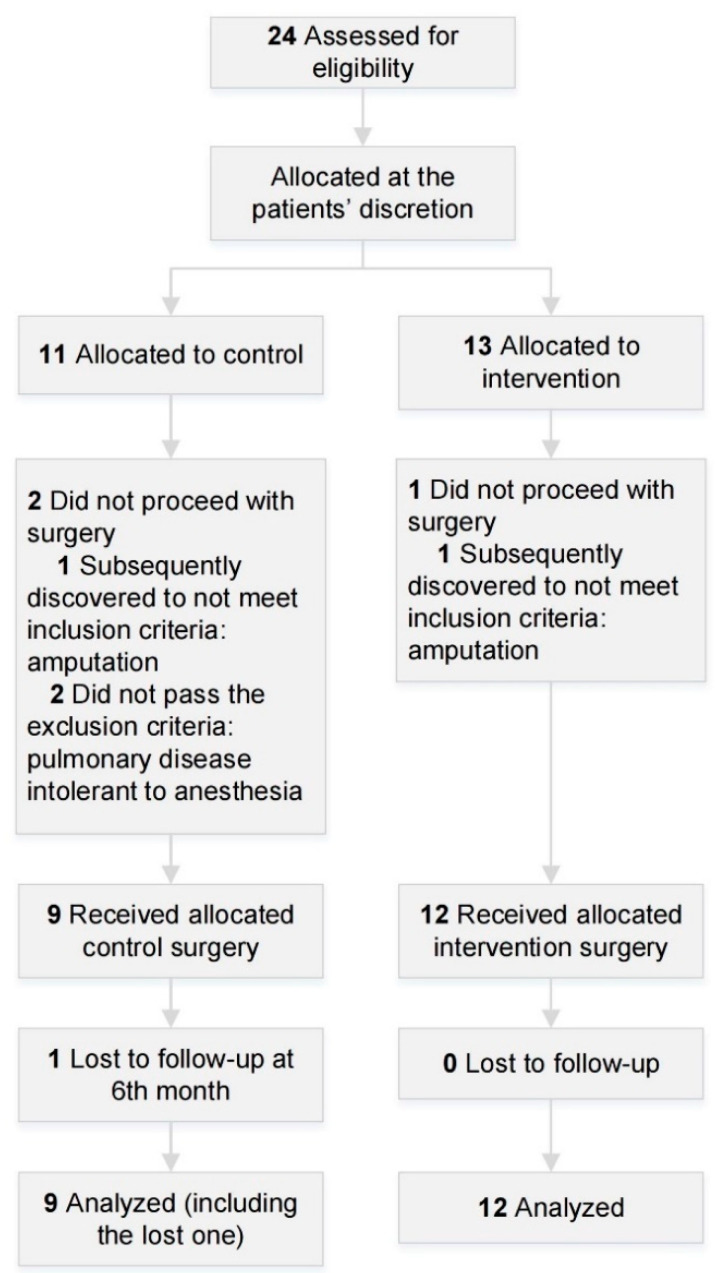
Flow of participants in the study of preoperative planning using 3D-multimodality imaging for soft tissue sarcoma of the axilla.

This prospective observational study compared preoperative planning based on 3DMMI to traditional images for the surgical treatment of axillary STS. The ethical committee of our institution approved the study protocol (No. 2019-1062). This study followed the Strengthening the Reporting of Observational Studies in Epidemiology reporting guidelines (Figure 1) and was conducted in compliance with the ethical standards of our hospital and the Helsinki Declaration.

Eligibility requirements included biopsy-proven, resectable axillary STS, tumour diameter > 5 cm, age > 14 years, and a clinical course aimed at curative limb salvage surgery. The exclusion criteria were severe systemic disease and intolerance to surgery. Informed consent was obtained from all participants or their guardians.

### 2.2. Allocation and Management

The patients were allocated to two cohorts (3DMMI and non-3DMMI groups) at their own discretion. Each patient was evaluated by a multidisciplinary team of pathologists, radiologists, orthopaedic oncologists, radiation oncologists, and medical oncologists. Based on the biopsy histotypes, preoperative chemotherapy was administered in selected cases. Preoperative radiation therapy was indicated if an oncologically appropriate surgical margin was difficult to obtain [14].

Preoperative local imaging, including contrast-enhanced CT angiography and MRI, and MRN, was performed for all patients. For contrast-enhanced CT angiography, a multi-slice helical CT (SOMATOM Definition Flash, Siemens Healthcare, Erlangen, Germany) scan was performed with a slice thickness of 1 mm to generate plain scan images and artery and venous phase images. For contrast-enhanced MRI, using a 3.0-T MR scanner (MAGNETOM Skyra, Siemens Healthcare, Erlangen, Germany) with an 8-channel receive coil, a conventional T1 VIBE enhanced sequence was performed to generate T1 enhanced images to assist the registration process. Then, a T2W turbo spin echo (TSE) sampling perfection with application-optimised contrasts using different flip angle evolution (SPACE) sequences in the same MR device was prescribed for MRN in a coronal plane with the following parameters: echo time (TE) 195 ms; repetition time (TR) 3500 ms; inversion time 220 ms; flip angle 120; matrix size 512 × 512; field of view (FOV) 384 mm; slice thickness 1 mm.

Preoperative planning and the definitive surgery were conducted by one of two surgeons (H.D. and C.T.) with over 15 years of experience in STS. In the 3DMMI group, the planning was conducted based on 3DMMI, whereas in the non-3DMMI group, it was conducted based on conventional planning using separate 2D or 3D images.

The intra- and postoperative treatment protocols were similar between groups. Gross dissection was conducted through normal tissue planes without tumour contamination, with reference to the preoperative planning. For tumours adjacent to bone, the periosteum was removed. For cortical or medullary invasion, the affected bone segment was resected or reimplanted after sterilisation. For tumours close to major vessels, the vessels were not resected if the underlying structures were grossly intact after adventitia removal [14]. Otherwise, major vessels were replaced with a great saphenous vein graft or artificial vessels. The major nerves were always retained, and the perineurium was removed if the nerves were immediately adjacent to the tumour.

Postoperative radiation therapy was indicated and recommended for patients with all positive surgical margins and stage II, IIIA, and IIIB extremity STS, according to the American Joint Committee on Cancer (AJCC) [14]. Chemotherapy was also considered for specific STS histotypes. The patients were followed up at two weeks and 1, 3, 6, and every 3 to 6 months thereafter in the outpatient department. Chest CT and axillary MRI were performed to detect lung metastasis and local recurrence, respectively.

### 2.3. Model Preparation and Use

In the 3DMMI group, MRI and MRN were mapped to CT in register using affine and diffeomorphic registration algorithms in advanced normalisation tools [11]. The tumour and adjacent critical structures were segmented with level-set, region-grow, and threshold control in 3D Slicer 4.11 (www.slicer.org, accessed on 20 March 2022) [15]. Quality assurance and manual correction were performed to achieve registration accuracy > 95% and a maximum segmentation error < 2 mm compared with the original DICOM data. The time spent on model preparation was recorded.

**Figure 2:**
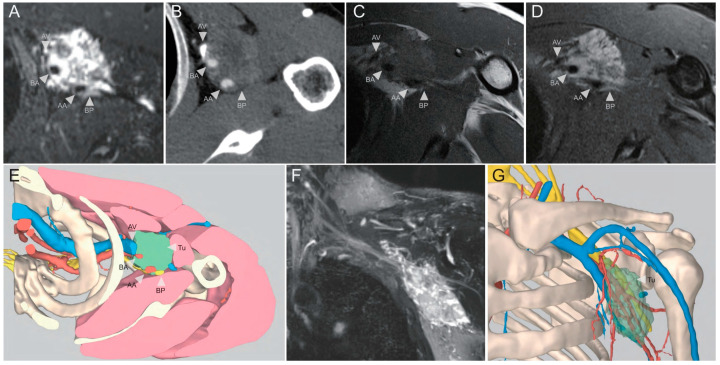
Classic images and their 3D reconstructions in a patient with axillary Ewing’s sarcoma. (**A**) MR hydrography image; (**B**) CT angiography image; (**C**) T1-weighted MRI; (**D**) T2-weighted MRI; (**E**) multimodality 3D reconstruction image with similar axial views as (**A**–**D**); (**F**) coronary view of MR hydrography image; (**G**) anterior view image of multimodality 3D reconstruction. Traditional surgical planning (control group) relies on classic images (**A**–**D**, **F**) alone, whereas the 3D multimodality image group has additional 3D reconstructions based on these classic images. Abbreviations: AV, axillary vein; BA, brachial artery; AA, axillary artery; BP, brachial plexus; Tu, tumour.

**Figure 3:**
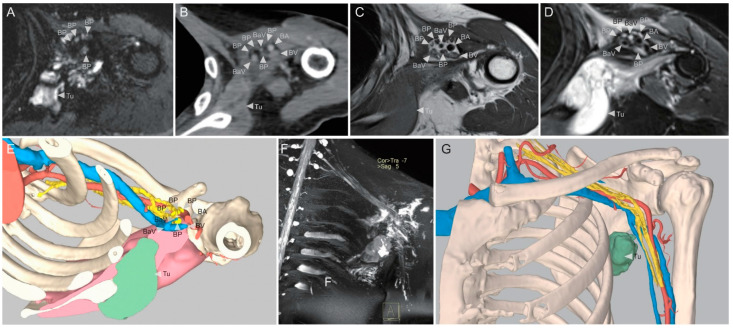
Classic images and their 3D reconstructions in a patient with liposarcoma. (**A**) MR hydrography image; (**B**) CT angiography image; (**C**) T1-weighted MRI; (**D**) T2-weighted MRI; (**E**) multimodality 3D reconstruction image with similar axial views as (**A**–**D**); (**F**) coronary view of MR hydrography image; (**G**) anterior view image of multimodality 3D reconstruction. Abbreviations: BA, brachial artery; BaV, basilic vein; BV, brachial vein; BP, brachial plexus; Tu, tumour.

The model included the bones (scapula, clavicle, humerus, and ribs), tumour, major neurovascular structures (at least those adjacent to the tumour), muscles (segmented as much as possible), enlarged lymph nodes (if any), and bone oedema (if any), which were reviewed by surgeons using smartphones or computers (Figure 2 and Figure 3).

### 2.4. Data Collection and End-Point Selection

The patient enrolment period was between November 2019 and December 2020, whereas data collection was from November 2019 to June 2021. Clinical baseline data included sex, age, tumour size, histology type, Fédération Nationale des Centres de Lutte Contre le Cancer (FNCLCC) grading, stage, and neurovascular involvement [16]. Tumour size was recorded as the maximum diameter according to the preoperative cross-sectional images. Neurovascular involvement was defined as >1/2 complete encasement as a preoperative image finding, whereas simple microvascular or perineural invasion identified in the final pathological specimen was excluded.

The primary endpoints were the surgical margins and complications. The surgical margins were first documented by surgeons and later confirmed by an expert sarcoma pathologist using the Toronto Margin Context Classification (TMCC) [17,18,19]. The margins were classified into four categories: negative margins (R0 according to AJCC residual tumour classification); inadvertent positive margins (IPMs); planned close, but with an ultimately positive microscopic margin along a major bone or neurovascular structure; and positive margins after a second resection for patients treated initially elsewhere with inadequate margins. Surgical complications included injury to neurovascular structures and wound complications. Vascular injury was defined as an intraoperative inadvertent major vessel injury necessitating suture repair, and nerve injury as new postoperative limb function morbidity.

The secondary endpoints were operative time, blood loss, serum index of systemic inflammation, length of hospital stay, and limb function. Operative time and blood loss were dichotomised as less or more than the 75th percentile in both. The serum index of systemic inflammation encompassed C-reactive protein (CRP) and interleukin–6 (IL–6) levels recorded preoperatively and two days postoperatively to reflect the magnitude of surgical trauma [20,21]. Limb function was assessed using the Musculoskeletal Tumour Society (MSTS) and the Disabilities of the Arm, Shoulder, and Hand (DASH) scores both preoperatively and at six months postoperatively [22,23].

### 2.5. Statistical Analysis

Sample size calculation was not performed due to the limited availability of previously published data. Continuous variables were tested for normality using the Shapiro–Wilks test and presented as means (standard deviation) if normally distributed and were otherwise expressed as medians (interquartile range). Categorical variables were presented as proportions (percentage). Between-group comparisons were assessed using the independent-sample t-test for continuous variables and the Mann–Whitney U test and Fisher’s exact test for categorical variables. R version 3.5.3 (R Foundation for Statistical Computing, Vienna, Austria) was used for the analyses; a two-sided *p* < 0.05 indicated statistical significance

### 2.6. Protocol Updates

This study was initially registered involving all bone and soft tissue tumours. Patients aiming for palliative surgery or amputation procedures were excluded to reduce heterogeneity in this report. The secondary indicator, tumour volume, was updated to the tumour size recorded at the maximum diameter during the first enrolment of the patient, since it was difficult to measure the volume in the non-3DMMI group. Performance status, another secondary indicator, was removed after the fifth enrolled case due to its low relevance.

## 3. Results

Between November 2019 and December 2020, 24 patients with axillary STS were identified, of which three were excluded (one had severe chronic pulmonary disease intolerant to anaesthesia and the other two underwent amputation after admission). Twenty-one patients (nine male, mean [standard deviation] age, 43.8 [16.0] years) were enrolled in this analysis, with nine in the non-3DMMI group and 12 in the 3DMMI group. The baseline characteristics indicating no statistical differences between groups are listed in Table 1.

There were no statistically significant differences in the overall surgical margins (*p* = 0.379), IPM (1/12 vs. 3/9, *p* = 0.272), intraoperative complications (*p* = 0.429), and postoperative wound complications (*p* = 0.788). Moreover, there were no significant differences ≥75th percentile vs. <75th operative time percentile (*p* = 0.331) and blood loss (*p* = 0.159), or second postoperative day serum CRP (*p* = 0.586) and IL-6 levels (*p* = 0.367) between the groups. Further, MSTS and DASH showed no statistically significant differences (*p* = 0.416, and *p* = 0.517, respectively) at six months postoperatively. There were significant differences in operative time (*p* = 0.048), blood loss (*p* = 0.038), and length of stay (*p* = 0.046) (Table 2). One patient in each group underwent major vessel replacement for underlying gross tumour involvement, and no clavicle osteotomies were performed. The median time spent on model preparation was 3.5 h (range, 3.0–4.0 h) per patient.

The median follow-up duration was 10 months (range, 6–18 months). One patient in the non-3DMMI group was lost to follow-up at six months postoperatively. No statistical differences were observed in MSTS (preoperative vs at six months) for the non-3DMMI (*p* = 0.063), 3DMMI group (*p* = 0.063), and DASH (*p* = 0.156, and *p* = 0.055).

## 4. Discussion

Axillary STS is challenging to resect because of its proximity to vital neurovascular bundles and occasional involvement. The literature related to axillary STS is limited since most studies have reported small cohorts of patients with STS in the axilla grouped with other locations [24,25,26,27,28]. Moreover, most of these studies emphasised patient demographics and treatment outcomes. However, this study explored ways to improve surgical outcomes from the perspective of preoperative planning based on multimodal images, which revealed these critical anatomical structures in 3D. The study found that 3DMMI can improve preoperative planning and surgical outcome for patients with axillary STS by reducing operative time, blood loss, and length of hospital stay and alleviating the risk of inadvertent residuals. Therefore, surgeons should consider 3D reconstruction from multimodal images when dealing with complicated STS, especially with neurovascular involvement.

A successful surgery depends mainly on the surgeon’s skills and knowledge of anatomy. While the skills may remain constant, the anatomy varies owing to innate or tumour-related variations. Although sarcoma specialists may accomplish a limb salvage STS surgery well, even with large unexpected intraoperative anatomical variations, less experienced surgeons are more likely to injure the nerves and vessels or have prolonged operative time, especially in highly complicated cases. Therefore, preoperative images are used to detect these potential unexpected variations and tumour surroundings. In standard preoperative planning, surgeons need to recognise key anatomical structures from each image and roughly form an image of the tumour in the brain, and if necessary, communicate with radiologists in words, which is the usual method of so-called transdisciplinary collaboration. Despite being technically more capable of reading images than surgeons, especially young ones, radiologists cannot describe the image in complete detail, since words are always less intuitive and informative than a 3D representation. Hence, in a conventional setting when relying on the rough classic tumour image, surgeons may still face significant uncertainty, with unexpected details uncovered intraoperatively. However, most potential challenges can be resolved preoperatively rather than as an unexpected intraoperative encounter, based on improved preoperative planning using 3DMMI processed by a radiologist. This 3D image serves as the best tool for connecting surgeons and radiologists and facilitates better transdisciplinary teamwork. Surgeons may easily devise an improved surgical approach with a more detailed knowledge of patient-specific 3D anatomy, including the tumour and all adjacent critical structures, resulting in a minimised risk of major neurovascular injury and less time spent in dissection.

Apart from providing comprehensive knowledge of patient-specific anatomy, 3DMMI could also help with tailored individualised preoperative planning that can hardly be done with conventional separate images or which can be done in only a very rough manner. While surgeons have been trained to mentally integrate anatomical structures from different imaging modalities, a fused digital display of multimodal images is far more informative and diagnostically reliable [29]. Surgeons could design a detailed 3D surgical approach with this digital 3DMMI, including through which layer of anatomical structure the dissection/resection should be performed, in which plane the dissection should be performed with an involved major nerve or vessels, whether to perform an osteotomy to help the resection, and the exact position for the osteotomy. A recent study by Sambri et al. observed an increased local recurrence risk for STS close to major vessels immediately adjacent to or surrounded by the tumour, as compared with indirect vascular proximity to the tumour, indicating individualised limb salvage procedures [30]. To provide better local control and limit unnecessary trauma, 3DMMI can intuitively and easily help identify the surgical needs of patients, that is, whether to preserve or resect a major vessel and the appropriate site/length of resection, if necessary. The aforementioned preoperative planning can be difficult even for some experienced STS specialists, whereas, in most cases, especially among young and inexperienced surgeons, the surgical plan is designed after visualising the tumour surroundings intraoperatively. Therefore, with 3DMMI, the incidence of local recurrence associated with poor preoperative planning and operative time can be decreased, particularly for less experienced surgeons.

To evaluate outcomes related to surgical planning, TMCC was used to assess the resection margin, as it has a unique role in IPM residuals [18]. In this study, the percentage of IPMs in the 3DMMI group was a quarter of that in the non-3DMMI group (1/12 vs. 3/9); however, the difference was not statistically significant. We presumed that this was due to the small sample size. Nevertheless, this pilot study provides a rational basis for future multi-centre trials.

Achieving local control cannot not solely define a successful limb salvage STS operation; surgical metrics are also important. Despite similarities in intraoperative complications, inflammation index, and limb function between the two groups, operative time and blood loss were significantly improved in the 3DMMI group. Since it was impossible to limit bleeding in the axillary region by a tourniquet, faster surgery with a clear surgical field was necessary. By revealing critical anatomical structures and their relationships in 3D, 3DMMI facilitated preoperative decisions may help minimise surgical challenges related to uncertain anatomical parameters, especially in highly complicated cases, resulting in reduced operative time and intraoperative blood loss. Additionally, a shorter length of hospital stay was also observed, which may limit various potential in-hospital complications and additional costs, and may save healthcare resources [31]. Therefore, preoperative planning based on 3DMMI is promising for successful STS surgery in this setting.

In addition to the potential benefits previously mentioned for patients and hospitals, 3DMMI-based preoperative planning may also facilitate a reduced cognitive load on surgeons by simply decreasing the amount of information processing required for both experienced and young surgeons [32]. Cross-sectional images, such as CT and MRI, depict almost every element of the subject’s anatomical information in grayscale, including structures irrelevant to surgical decision-making. Extraneous information may affect surgeons’ cognition when interpreting imaging. Conversely, redundant structures that are less relevant to surgery are excluded from the 3DMMI representation. The vital structures and their spatial relationships are highlighted and visualised in 3D and colour. Thus, surgeons can focus on devising a personalised surgical plan with ease.

Even though 3DMMI features the advantages discussed above, the deviation between the medical images and the real anatomy of the human body should not be overlooked. Although 3D images may conform to the preoperative images, the different patient’s positioning between preoperative imaging and surgery results in a 3D shift of all anatomical structures, especially in the lower limbs. The mean femoral artery shift is 3.28 mm for repeated supine positions and 14.72 mm for supine vs. right lateral decubitus position in the thigh, whereas the displacement of the tibial nerve is much less in the lower leg, ranging from 0.9 to 3.02 mm for all positions [7]. Although the sarcoma-involved neurovascular structures may stay in a relatively constant spatial location with the tumour, it is still recommended to capture preoperative images in the same position as the surgery to reduce the variability, especially when using 3DMMI images for surgical planning for STS from less rigid compartments.

Another issue that should be addressed is the time spent in 3D imaging processing. In this study, the median time was 3.5 h per patient even with semi-automatic algorithms including level-set, region-grow, and threshold control, which is much longer than writing a conventional medical image diagnostic report. However, it was conducted in the image post-processing Centre in our hospital instead of by a single radiologist from the conventional radiology department. We believe that the time spent was highly acceptable considering its benefits to patients, and potential value to personalised radiotherapy planning, rehabilitation procedures, and doctor–patient communication. Nevertheless, automatic algorithms are needed to reduce the time spent for 3D processing in the future.

Our study had some limitations. First, the sample size was small and uncalculated due to limited previously published data, and groups were heterogeneous with different histologies and grades. However, this was a prospective preliminary pilot study exploring the potential of a novel imaging technique for preoperative planning for a group of rare tumours encompassing over 100 histologic subtypes in unusual and challenging locations, which may lay the foundation for our future large-scale trials. Second, the follow-up period was relatively short. Nevertheless, the outcome measures were intraoperative findings and early clinical outcomes, which required a limited follow-up duration. However, follow-up on these patients will be continued and a standard survival analysis with special attention to local recurrence will be performed in the future. Third, the surgical margin was used to assess local control in this study, whereas the correlation between margin and local recurrence remains controversial, particularly in some specific STS histotypes with infiltrative growth patterns, such as myxofibrosarcoma and undifferentiated pleomorphic sarcoma [33,34,35,36]. Additionally, the “optimal” margin and its width have been debated in the literature [37]. However, the principal purpose of this preliminary study is to present a potentially useful 3D image-based tool. This may facilitate preoperative planning procedures and minimise unexpected surgical challenges related to neurovascular injury and IPM by giving comprehensive knowledge of patient-specific anatomy. This pilot study did not aim to directly establish correlations between 3DMMI and local recurrence. Nevertheless, our future large-scale trial will focus on local recurrence rather than on surgical margins only. Four, since the surgeries in this study were performed by 15-year-experience experts, the outcome measures and strength of 3DMMI might be underestimated when used by less experienced surgeons.

## 5. Conclusions

In summary, this study reported a real-world institutional experience of 3DMMI-based preoperative planning for patients with axillary STS, where different images synergistically contributed to more comprehensive guidance for surgical planning in three dimensions, especially for less experienced surgeons. These findings, while still preliminary, may affect the performance of preoperative planning for axillary STS. Furthermore, this planning may benefit patients, surgeons and hospitals in various ways. Improved outcomes for the patients were found, such as less intraoperative blood loss, shorter operative time, and a potentially lower risk of inadvertent residuals. The use of 3DMMI may also provide an intuitive and easy approach to improving surgical planning for surgeons, and hospitals would benefit from a shorter operative time and length of hospital stay. Overall, this pilot study yielded promising results that will support further studies in the future.

## Figures and Tables

**Table 1:** Baseline characteristics between groups who underwent limb salvage surgery with and without 3DMMI-based preoperative planning.

Characteristic		Participants, No. (%)		*p* Value
		Non-3DMMl (*n* = 9)	3DMMI (*n* = 12)	
Age, mean (SD), y		45.2 (18.8)	42.8 (14.3)	0.736 ^d^
Male				>0.999 ^e^
	Male	4 (44)	5 (42)	
	Female	5 (56)	7(58)	
Histological type				0.885 ^e^
	Synovial sarcoma	2 (22)	3 (25)	
	Liposarcoma	2 (22)	3 (25)	
	Rhabdomyosarcoma	2 (22)	2 (17)	
	Undifferentiated pleomorphic sarcoma	1 (11)	2 (17)	
	Angiosarcoma	1 (11)	0	
	Ewing	0	1 (8)	
	Malignant fibrous histiocytoma	1 (11)	1 (8)	
FNCLCC grade				0.904 ^f^
	1	1 (11)	1 (8)	
	2	3 (33)	4 (33)	
	3	5 (56)	7 (58)	
Tumour Size ^a^, median (interquartile range), cm		8 (5.0)	8 (4.8)	0.902 ^f^
Neurovascular Involvement				
	Vessel	4 ^b^ (44)	4 ^c^ (33)	0.673 ^e^
	Nerve	5 ^b^ (56)	5 ^c^ (42)	0.670 ^e^
Tumour Stage				0.864 ^f^
	ⅠB	1 (11)	1 (8)	
	ⅢA	6 (67)	8 (67)	
	ⅢB	2 (22)	3 (25)	

^a^ Tumour size was recorded at maximum diameter; ^b^ three patients had both vessel and neural involvement; ^c^ three patients had both vessel and neural involvement; ^d^ Student’s T-test; ^e^ Fisher’s exact test; ^f^ Wilcoxon rank sum test.

**Table 2:** Comparative outcomes between groups who underwent limb salvage surgery with and without 3DMMI-based preoperative planning.

Outcome			Non-3DMMl (*n* = 9)	3DMMI (*n* = 12)	*p* Value
Surgical margin, No. (%)					0.379 ^a^
		Negative	3 (33)	5 (42)	
		Planned close	3 (33)	6 (50)	
		IPM	3 (33)	1 (8)	0.272 ^b^
Surgical complications					
		Intraoperative, vascular injury	3	1	0.429 ^b^
		Intraoperative, nerves injury	0	0	
		Postoperative, wound complication	0	1	0.788 ^b^
Operative time, mean (SD), min					
		Mean (SD)	134.4 (35.75)	101.7 (34.60)	0.048 ^c^
		Median (IQR)	120 (60)	95 (37.5)	
		≥75th percentile, participants, No. (%) ^d^	4 (44)	2 (17)	0.331 ^b^
Blood loss (mL)					
		Mean (SD)	338.9 (89.4)	258.3 (76.4)	0.038 ^c^
		Median (IQR)	350 (150)	250 (100)	
		≥75th percentile, participants, No. (%) ^d^	5 (56)	2 (17)	0.159 ^b^
Serum index of systemic inflammation					
		CRP, preoperative, median (IQR), mg/L	3.77 (2.00)	3.99 (1.52)	0.602 ^a^
		IL-6, preoperative, median (IQR), pg/mL	2.56 (1.49)	2.86 (1.67)	0.754 ^a^
		CRP, at 2nd day, mean (SD), mg/L	40.11 (13.97)	37.63 (6.05)	0.586 ^c^
		IL-6, at 2nd day, mean (SD), pg/mL	36.56 (11.98)	32.89 (5.98)	0.367 ^c^
Length of in-hospital stay, median (IQR)			8(1.5)	7(1)	0.046 ^a^
Limb function					
		MSTS, preoperative, median (IQR)	90 (13)	93 (9.3)	0.270 ^a^
		DASH, preoperative, median (IQR)	8 (18)	7.5 (10)	0.430 ^a^
		MSTS, at 6th month, median (IQR)	93 (8.5)	95 (7.0)	0.416 ^a^
		DASH, at 6th month, median (IQR)	8 (9)	5.5 (8.75)	0.517 ^a^

^a^ Wilcoxon rank sum test; ^b^ Fisher exact test; ^c^ Student’s T-test; ^d^ 75th percentiles are defined as 150 min for operative time and 350 mL for blood loss. Abbreviations: IPM, inadvertent positive margins; CRP, C-reactive protein; IL-6, interleukin–6.

## Data Availability

The data presented in this study are available on Zenodo (https://doi.org/10.5281/zenodo.6471584). The corresponding authors will grant readers access to these data on reasonable request.
